# Identification of antigenic linear peptides in the soil-transmitted helminth and *Schistosoma mansoni* proteome

**DOI:** 10.1371/journal.pntd.0009369

**Published:** 2021-04-28

**Authors:** Johnny Vlaminck, Ole Lagatie, Daniel Dana, Zeleke Mekonnen, Peter Geldhof, Bruno Levecke, Lieven J. Stuyver

**Affiliations:** 1 Department of Virology, Parasitology, Immunology and Physiology, Ghent University, Merelbeke, Belgium; 2 Global Public Health R&D, Janssen Pharmaceutica NV, Beerse, Belgium; 3 School of Laboratory Science, Faculty of health science, Institute of health, Jimma University, Jimma, Ethiopia; National University of Ireland Galway, IRELAND

## Abstract

The scientific community identified non stool-based biomarkers as the way forward to support soil-transmitted helminth (STH; *Ascaris lumbricoides*, *Trichuris trichiura* and the hookworms *Ancylostoma duodenale* and *Necator americanus*) and schistosome (*S*. *mansoni* and *S*. *haematobium*) deworming programs. This support is needed in making the decision of whether or not to stop preventive chemotherapy intervention efforts and to ultimately transition towards a post-intervention surveillance phase. We applied a two-step micro-array approach to identify antigenic linear epitopes in the STH and *S*. *mansoni* proteomes. In a first experiment, we identified antigenic peptides by applying sera from 24 STH and/or *S*. *mansoni* infected Ethiopian children on a high-density peptide microarray containing 3.3 million peptides derived from the complete STH and *S*. *mansoni* proteomes. A second array experiment with 170,185 peptides that were recognized in the first array was designed to identify non-specific antibody reactivity by applying sera from 24 healthy individuals from Belgium (a non-endemic country). From this array testing cascade, several peptides were identified for STH but none of them appeared to be unique for one species. We therefore concluded that for STH, none of the peptides revealed to be sufficiently sensitive or species specific. For *S*. *mansoni*, some promising peptides were identified prompting future investigation. Based on these results, it is unlikely that linear epitopes would be highly useful in detecting species-specific antibody responses to STH in endemic communities. For *S*. *mansoni*, one particular peptide of the micro-exon gene 12 (MEG-12) protein deserves further research. In addition, this study emphasizes the need of well-characterized biobanks for biomarker discovery, particularly when the integration of multiple disease programs is envisioned.

## Author summary

Today, infections with intestinal (roundworms, whipworm and hookworms) and blood-dwelling worms (schistosomes) are traditionally diagnosed by demonstrating worm eggs in stool. This current practice comes with some important challenges, including but not limited to the low-throughput and the need of skilled operators. Especially in the context of monitoring and evaluation, there is a need for alternative tools. Therefore, one of the recommendations of the scientific community was to develop tests that are based on the detection of antibodies in blood, which reflect the natural immune response of the host to worm-specific components or antigens. In the present study, we screened the antigenicity of all the peptides that build up the proteome of intestinal and blood-dwelling worms. For intestinal worms, our results revealed that the antibody response to these peptides was either not unique for the worm species or absent in infected subjects. For blood-dwelling worms, the findings were less sobering, with a number of peptides inducing an antibody response that was only observed in infected subjects.

## Introduction

Nearly two decades ago, during the 54^th^ World Health Assembly, resolution WHA54.19 was approved. This resolution urged member states to increase efforts to control two of the most impactful neglected tropical diseases (NTDs), namely soil-transmitted helminthiasis (caused by *Ascaris lumbricoides*, *Trichuris trichiura* and the hookworms *Ancylostoma duodenale* and *Necator americanus)* and schistosomiasis (predominantly caused by *Schistosoma mansoni* and *S*. *haematobium*) by ensuring access to essential drugs. Combining their burden of disease, they affect an estimated 1 billion individuals worldwide, resulting in a health toll of over 3.3 million daily adjusted life years [1,2].

Periodic administration of anthelmintic drugs to at-risk populations has long been a cornerstone in the control of both soil-transmitted helminth (STH) and *Schistosoma mansoni* (SCH) infections. In 2012, continued support for control efforts and drug availability was stimulated and safeguarded with the development of the NTD roadmap for STH and SCH infections and the London declaration on NTDs [3]. For example, treatment coverage in school-aged children (SAC) for STH and SCH infections has since then increased from approximately 29% and 23% to 60% and 61% in 2018, respectively [4]. The recently published NTD roadmap for 2021–2030 reflects the continued ambition to continue the fight against these NTDs [5], with the specific aim of reaching elimination of soil-transmitted helminthiasis and schistosomiasis as a public health problem in 96% and 100% of the endemic countries, respectively. However, in order to monitor whether programs have reached these ambitious targets, the development and availability of sensitive and specific diagnostics is critical.

Today, standard diagnosis of STH and SCH infections is made by examining stool for the presence of helminth eggs using the Kato-Katz method (STH and *S*. *mansoni* infections) or by urine filtration (*S*. *haematobium*) [6,7]. Although both these methods are relatively simple and cheap, they are labor intensive, depend on human interpretation of the results (i.e. skills to recognize eggs in sample), have a limited sensitivity to detect low-intensity infections [8,9] and come with significant logistical challenges associated with sample collection and turnaround time (sample should be examined as soon as possible after collection). Therefore, as programs move towards a verification of elimination as a public health problem and examine the prospects of breaking disease transmission [10,11], the usefulness of these traditional methods is questionable. The use of other, sensitive and specific, high-throughput diagnostic options will be needed to measure progress towards WHO goals.

In 2018, the STH community produced target product profiles (TPPs) for each of the four different phases of a control program (use-case 1: determine disease transmission and identify type of mass drug administration (MDA), use-case 2: assess progress against program goals, use-case 3: confirm a decision to stop intervention and transition into surveillance, and use-case 4: verify a sustained break in transmission [12]). It was concluded that a diagnostic biomarker for implementation toward the later stages of a control program (use case 3&4) should be present and detectable in a readily accessible body fluid, excluding stool [12,13]. Moving away from stool as a diagnostic matrix provides some advantages including (i) increased sample throughput (will be necessary as sample sizes increase), (ii) improved compliance rates, especially in populations older than SAC and woman of child-bearing age, (iii) higher confidence regarding sample origin (collection on the spot, no stool exchanges or contaminations possible), (iv) no personnel and environmental contamination during field collection, lab analysis and discarding the leftover stool samples, (v) use of methods with higher sensitivity to detect parasite exposure compared to the detection of parasite eggs or DNA in the stool (which does not always accurately reflect exposure status [14], especially in low endemic settings) and (vi) finally, it would pave the way to a more integrated NTD control program, combining serological markers for multiple other co-endemic NTDs (e.g. lymphatic filariasis, onchocerciasis, and strongyloidiasis) for which serum-based diagnostic solutions already exist and are being used [15–17]. To date, the community lacks appropriate tools to confirm a break in transmission both for STH infections. For SCH the introduction of lateral flow tests detecting *Schistosoma* gut-associated polysaccharides (POC-CCA and POC-CAA) has shown great promise but especially in low endemic regions its accuracy has been questioned [18–20].

With the availability of parasite genomes and transcriptomes and the advances in high throughput technologies (e.g. microarray), it is now possible to screen for epitopes in numerous antigenic targets at once or even in complete proteomes [21–23]. In recent years, immunomics-based studies have identified novel antibody targets for schistosomiasis [24–25], hookworm [26] and other NTDs [21] by screening a pre-selected number of target proteins. However, scanning the complete proteome of any STH or SCH species has so far not been performed. The aim of this study was therefore to identify antigenic linear epitopes in the complete STH and *S*. *mansoni* proteome by performing two consecutive microarray experiments. The identified peptides could eventually support the development of novel diagnostic assays to be implemented in the different stages of STH and SCH control programs.

## Materials and methods

### Ethics statement

Human serum samples from Ethiopia were collected as part of a serological and parasitological screening in SAC and adults from Jimma, Ethiopia [27]. For the collection of stool and blood samples, ethical approval was obtained from the Institutional Review Board of both Ghent University, Belgium (reference number: 2015/0801 and study registration number: B670201526293) and Jimma University, Ethiopia (reference number: RPGC/181 and IHRPGD/680). Written informed consent was obtained from the parent/guardian of each participant under 18 years of age.

Plasma samples from Belgian healthy controls were collected as part of a biobank study in Beerse, Belgium [28–32]. The Ethics Committee of “ZiekenhuisNetwerk Antwerpen” and the ethics committee of the Antwerp University Hospital approved the protocol. Written informed consent was obtained from all individuals, and all samples were decoded and de-identified before they were provided for research purposes.

**Fig 1:**
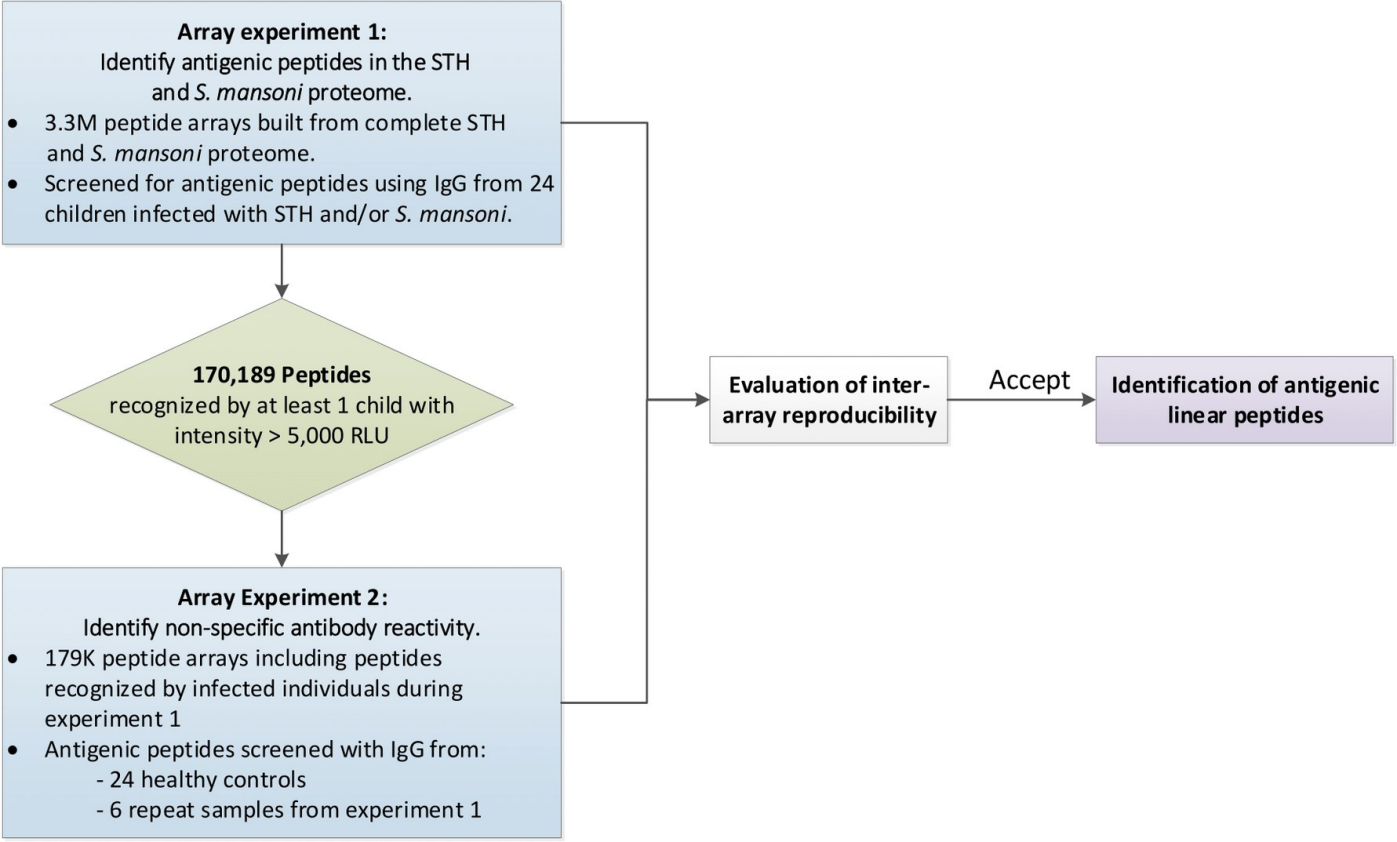
Overview of the different operational steps. (STH = soil-transmitted helminth, RLU = relative light units).

In this manuscript we describe the results of a two-step micro-array approach to identify antigenic linear epitopes in the complete STH and SCH proteomes. **Fig 1** provides a schematic overview of the experiments described in this manuscript. In a first array experiment (**array experiment 1**), we identified antigenic STH and SCH peptides by applying sera from 24 STH and/or SCH infected children on a high-density peptide microarray containing 3.3 million peptides derived from the complete STH and SCH proteomes. In a second array experiment (**array experiment 2**), we identified any non-specific antibody reactivity. For this, we applied sera from 24 healthy individuals from Belgium (a non-endemic country for STH/SCH) on an array containing a subset of 179 thousand peptides that were detected in the array experiment 1 by at least one individual. To assess the inter-array reproducibility, six samples were included in both experiments. Following the assessment of the reproducibility across arrays, we developed a final list of antigenic linear peptides for each of the different helminth species.

### Array experiment 1: Identify antigenic peptides in the STH and *S*. *mansoni* proteome

#### Array design and synthesis

A total of 110,208 predicted protein sequences were used for array design. These data were obtained from WormBase ParaSite [33]. **Table 1** provides more details on the used protein databases. A list of 2,832,723 peptides (13- to 16-mers) was generated from these proteins, by tiling all proteins using a 5-residue offset. A total of 165,854 peptide sequences from the worm proteomes were considered redundant as they were either repeated within the same organism or found across different organisms.

**Table 1:** Overview of STH and *S*. *mansoni* proteome databases used for array design. Databases were obtained from the Wormbase Parasite repository in March 2017 [33].

Organism	Reference genome	Number of predicted proteins	Total protein length (amino acids)
*Ascaris lumbricoides*	PRJEB4950 v1.5.4	23,604	7,164,414
*Ascaris suum*	PRJNA80881 v1.0	18,542	6,058,746
*Trichuris trichiura*	PRJEB535 v2.1	9,650	4,186,051
*Ancylostoma duodenale*	PRJNA72581 v2.2	27,485	5,832,435
*Necator americanus*	PRJNA72135 v1	19,153	5,136,657
*Schistosoma mansoni*	PRJEA36577 v5.2	11,774	5,608,274
***Total***		***110*,*208***	***33*,*986*,*577***

The array also included control peptides, including a scramble peptide (WTKTPDGNFQLGGTEP) [34], one John Cunningham virus peptide (JCV: NLVRDDLPALTSQEI) [31] and two Epstein-Barr viral peptides (EBV1: PGRRPFFHPVGEADY and EBV2: FHPVGEADYFEYHQE) in 135-flold. The scramble peptide was included to confirm that the arrays did not detect a response for such a non-relevant peptide (i.e. average signal for this scramble peptide <5,000 relative light units (RLU) for all samples). Viral peptides were included to determine whether the arrays could detect an immune response against these viral peptides (i.e. average signal for this viral peptide >5,000 RLU) at a frequency similar to the seroprevalence described in literature for these viruses (82–94% for EBV and 64% for JCV [31,35]). Additionally, 2,220 peptides that originated from the *Onchocerca volvulus* proteome were included in 4-fold. Half of these peptides (1,110) were not recognized by *O*. *volvulus* infected individuals. The other 1,110 peptides included those against which the highest reactivity in *O*. *volvulus* patients was measured on a similar immune-array previously performed [23]. These *O*. *volvulus* peptides were included to assess the antigenicity of these peptides in a tropical, but non-*Onchocerca* endemic population.

Each peptide microarray slide (Roche Nimblegen) represented an entire set of peptides and one array per sample was used for screening. Peptides were synthesized in situ from C- to N-terminus and Serine was added N-terminally for improved solubility to conduct serum IgG profiling (NxT-Dx, Vienna, Austria). A list of all peptides, including their sequence and position on the arrays is available at https://osf.io/q5s9y/?view_only=8a464d9eafe6444b83b967a11f3211cd.

#### Selection of serum samples

For the first array experiment, a panel of 24 serum samples from Ethiopian SAC with known STH and *S*. *mansoni* infections were selected. **Table 2** provides an overview of the samples and their parasitological measurements. Donated stool samples were analyzed by three copromicroscopic methods to reduce the chance of false negative samples. Each sample was analyzed by single Kato-Katz, McMaster and Mini-FLOTAC method according to standard protocols [6,36,37]. Additionally, serum IgG4 response to *Ascaris* lung stage L3 larval extract was analyzed as previously published [27]. Thus, only *Ascaris* EPG positive children that showed an *Ascaris* ELISA optical density ratio (ODr) over the cut-off for positivity of 0.08 were considered for inclusion as *Ascaris* positive samples. Samples that were copropositive for *T*. *trichiura* and hookworm or *S*. *mansoni* had to show negative *Ascaris* ELISA values (<0.08 ODr) before being included in the sample set. Antibody responses to other helminth antigens were not determined. A total of 10 samples were positive for *A*. *lumbricoides*, 11 for *T*. *trichiura*, 9 for hookworm and 7 for *S*. *mansoni*.

**Table 2:** Characteristics of the sera used to screen peptide microarrays. Fecal egg counts (FEC; expressed as eggs per gram of stool (EPG) were based on a single stool sample. For STH, they represent the average FECs across single Kato-Katz, McMaster and Mini-FLOTAC method, whereas for *S*. *mansoni*, they represent the FEC based on a single Kato-Katz. For the *Ascaris* LungL3 IgG4 ELISA test, samples with an optical density ratio (ODr) of at least 0.08 were considered positive [27]. The grey cells indicate a positive test result for a given helminth or diagnostic test. Samples with an asterix (*) were included in both array experiments.

			Fecal egg count (EPG)				*Ascaris* LungL3 IgG4 ELISA (ODr)	Group
Sample ID	Age	Sex	*A*. *lumbricoides*	*T*. *trichiura*	hookworm	*S*. *mansoni*		
2015_287	6	F	1,626	0	0	0	0.147	Al
2015_330*	14	F	545	0	0	0	0.428	Al
2015_129	8	M	16	0	0	0	0.613	Al
2015_218	14	F	34,699	1,523	0	0	1.063	Al+Tt
2015_268	14	F	1,715	496	0	0	0.664	Al+Tt
2015_229	17	F	120	0	1,773	0	1.266	Al+Hw
2015_293	9	M	3,565	0	403	0	0.102	Al+Hw
2015_234	15	M	331	0	53	0	0.409	Al+Hw
2015_132	9	M	24	0	0	480	0.433	Al+Sm
2015_499*	15	M	17,500	0	0	360	0.045	Al+Sm
2015_587*	5	M	0	5,389	0	0	-0.057	Tt
2015_579	6	M	0	680	0	0	-0.126	Tt
2015_285	9	F	0	582	0	0	-0.106	Tt
2015_334	9	F	0	436	357	0	-0.055	Tt+Hw
2015_537	6	F	0	119	1,005	0	-0.109	Tt+Hw
2015_447	18	F	0	236	177	0	-0.079	Tt+Hw
2015_173	14	M	0	69	0	1,296	-0.030	Tt+Sm
2015_180	15	M	0	93	0	144	-0.041	Tt+Sm
2015_549*	16	M	0	643	0	528	-0.133	Tt+Sm
2015_335	8	F	0	0	556	0	-0.057	Hw
2015_368	18	M	0	0	122	0	-0.010	Hw
2015_48*	14	M	0	0	268	0	-0.050	Hw
2015_338*	8	F	0	0	0	1,248	-0.098	Sm
2015_341	9	M	0	0	0	1,128	-0.006	Sm

#### Serum sample processing

IgG from the serum samples was prepared by a simple spin-column purification procedure using Melon gel (Pierce, cat nr: 45206). Briefly, 15 μL of serum was diluted in 95 μL of Melon gel purification buffer, loaded onto Melon gel spin columns and centrifuged. The flow-through, containing pure IgG was collected and IgG concentrations were measured. Standardized IgG concentrations (0.025 mg/mL; final dilution in BLOTTO based binding buffer) of purified IgG were used for microarray analyses.

#### IgG microarray analysis

Microarray experiments were performed by NXT-Dx (NxT-Dx, Vienna, Austria). In brief, microarrays were washed once in TBST (Tris-Buffered Saline, 0.05% Tween 20), four times in TBS (Tris-Buffered Saline) and once in H_2_O. Microarrays were incubated at 4°C overnight with 5 μL of purified IgGs (final concentration 0.1 mg/mL) in 1% Alkali-soluble Casein in TBST. The microarrays were washed three times with TBST and incubated with secondary antibody (Alexa Fluor 647-AffiniPure Goat Anti-Human IgG, Fcγ Fragment Specific, Jackson 109-605-098) diluted 1:10,000 in 1% Alkali-soluble Casein TBST for 3 hours at room temperature. Finally, microarrays were washed three times in TBST, once in H_2_O and dried. Microarray fluorescent-signals were extracted from microarray images, and data sets were subjected to further statistical analysis in order to identify immunoreactive peptides.

### Array experiment 2: Identify non-specific antibody reactivity

Based on the results obtained from array experiment 1, a second, 179K peptide array was designed. A total of 170,185 peptides (13- to 16-mers) that presented signals of at least 5,000 RLU in at least one of the 24 samples screened on array experiment 1 were included in a smaller, follow-up array (array experiment 2). The threshold of 5,000 RLU was chosen based on previous work using the same type of peptide arrays [23]. A conservative selection (only 1 positive sample was sufficient to retain the peptide) was used because of the limited sample size and to ensure inclusion of all possibly antigenic peptides.

#### Array design and synthesis

Except for the absence of the two influenza hemagglutinin peptides, other control peptides were the same as described in array experiment 1. Peptide microarray slides (Roche Nimblegen) were divided into 12 subarrays, each presenting the entire set of peptides. One subarray per sample was subsequently used for screening. A list of all peptides, including their sequence and position on the arrays is available at https://osf.io/q5s9y/?view_only=8a464d9eafe6444b83b967a11f3211cd.

#### Selection of serum samples

A total of 30 samples were run on this second array. These included a panel of 24 plasma samples from Belgian healthy controls and six samples that were also used in the previous array (array experiment 1) in order to evaluate inter-array reproducibility of the results (these six samples are indicated in **Table 2**). Since Belgium is a non-endemic country for STH and SCH, it was assumed Belgian healthy controls were not exposed to STH or SCH. Serum sample processing and IgG microarray analysis were performed identical as during array experiment 1.

### Evaluation of inter-array reproducibility

To compare antibody responses measured in the 3.3M array and the 179K array, the data from both arrays were normalized together. Quantile normalization on base-2 log transformed data was used to correct for technical differences between arrays. Reproducibility between the two experiments was assessed for each of the six samples that were included on both arrays by correlating peptide specific antibody reactivity measured on the arrays. Correlations were performed using Spearman correlation. Statistical analysis was performed in R [38]. Both arrays were considered to be moderately or highly correlated in case Spearman’s correlation coefficient is >0.5 or >0.7, respectively [39,40].

### Identification of antigenic linear peptides

In order to identify antigenic parasite-specific peptides, for each peptide the number of positive samples (“positive” is defined as signal >5,000 RLU) was determined. Peptides were considered to be antigenic when (i) at least one of the specific helminth infected individuals was positive, and when (ii) not more than 1 out of 24 (i.e. <4.2%) healthy controls was positive. For peptides that fulfilled these two criteria, we calculated the average response of the infected group minus the average response of the healthy controls expressed in RLUs (= the Δ-value). Finally, the set of peptides was ranked based on this Δ-value to identify promising linear peptides.

### Peptide ELISA

Peptide ELISA’s were performed as described before [23,41–43].

## Results

### Array experiment 1: Identify antigenic peptides in the STH and *S*. *mansoni* proteome

We produced an array that contained 2,832,723 peptides representing a total of 110,208 protein sequences across the six different helminth species (**Table 1**) (*A*. *lumbricoides*, *A*. *suum*, *T*. *trichiura*, *A*. *duodenale*, *N*. *americanus*, *and S*. *mansoni*). These arrays were used to assess the IgG responses in 24 STH and/or *S*. *mansoni* infected individuals (**Table 2**).

**Fig 2:**
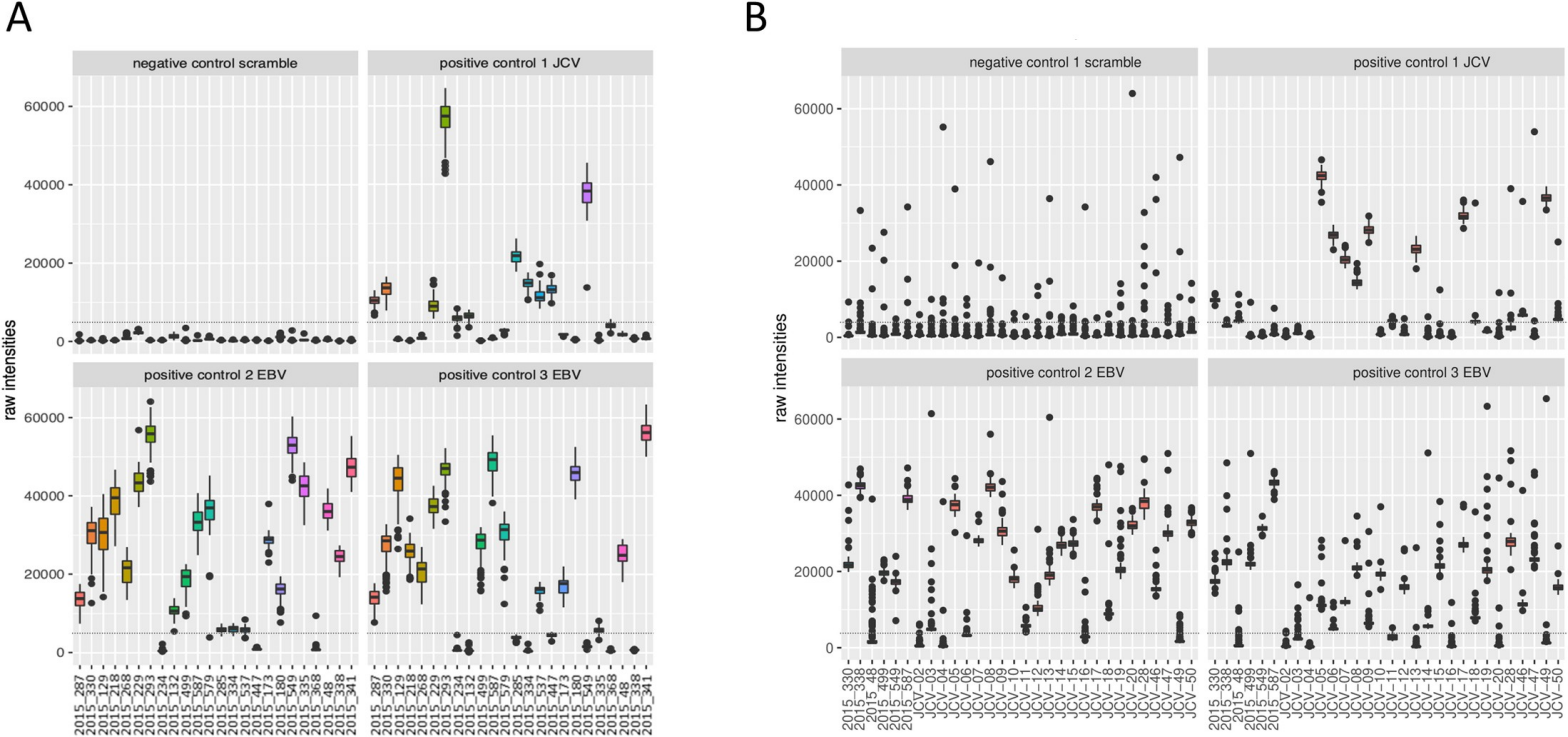
Box plots of raw intensities measured against control peptides in both microarray experiments. This figure represents the raw output values of IgG reactivity (y-axis) of the different samples (x-axis) to the different control peptides during array experiment 1 (**A**) and array experiment 2 (**B**). Both arrays included a scramble peptide (negative control), one John Cunningham virus (JCV) peptide and two Epstein-Barr viral peptides (EBV1 and EBV2) in 135-flold. Dotted line indicates the cut-off for positivity (5,000 relative light units (RLU)).

**Fig 3:**
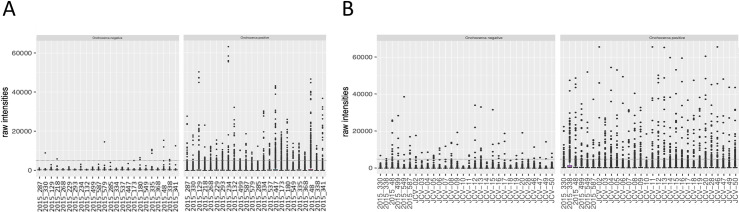
Box plots of raw intensities measured against *Onchocerca volvulus* peptides in both microarray experiments. **This** figure represents the raw output values of IgG reactivity (y-axis) of the different samples (x-axis) to the 1,110 negative and 1,110 positive *O*. *volvulus* peptides included on the microarray during both array experiment 1 (**A**) and array experiment 2 (**B**). Dotted line indicates the cut-off for positivity (5,000 relative light units (RLU)).

Even though there was some variability between sample reactivity to the negative scramble peptide, none of the samples showed an outlying signal above the threshold (5,000 RLU). Most of the subjects had high antibody responses against the two Epstein-Barr viral peptides (20 out of 24 for EBV1 and 16 out of 24 for EBV2). Fewer subjects reacted to the one John Cunningham virus peptide (JVC; 11 out of 24). Both the EBV and JCV peptides were comparable to the described prevalence of 82–94% and 64%, respectively (**Fig 2A and S1 Info**)). Non-reactive *O*. *volvulus* peptides showed very low reactivity, with only 1 peptide reacting consistently in 1 sample (OVOC5335; 523), indicating that these non-reactive *O*. *volvulus* peptides are also non-reactive in this non-*Onchocerca* endemic population. The highly reactive *O*. *volvulus* peptides were mostly negative, but some peptides were consistently found to be positive in one or more samples (**Fig 3A and S1 Info**).

Based on the data obtained from the 3.3M peptide arrays, a set of antigenic linear peptides was selected by filtering all peptides for which at least one sample had a signal above a threshold of 5,000 RLU. This resulted in a list of 170,185 peptides, comprising a total of 33,890 peptides that were selected from the proteome of *A*. *lumbricoides*; 29,424 from *A*. *suum*; 20,021 from *T*. *trichiura*; 28,669 from *A*. *duodenale*; 25,765 from *N*. *americanus* and 32,416 from *S*. *mansoni*. Of these 170,185 peptides, 150,451 peptides were unique, while 19,734 were present in more than one proteome.

### Array experiment 2: Identify non-specific antibody reactivity

A second array was produced with the set of 170,185 peptides that were recognized (produced a signal >5,000 RLU) by at least one of the STH and/or *S*. *mansoni* infected children included in array experiment 1. On this second array, the IgG responses of 24 healthy control samples from Belgium to this set of peptides were assessed. Additionally, six samples were included that were also included in array experiment 1 to assess the reproducibility across both arrays (samples included in both arrays are indicated in **Table 2**). Similar to the 3.3M arrays, none of the analyzed samples showed an average signal for the negative control peptides above the threshold of 5,000 RLU, but some outliers were observed in the replicates (76 out of 2,880, i.e. 0.026%), indicating slightly increased variability on detection of non-antigenic peptides in the second array. Most of the subjects showed high IgG reactivity against the EBV peptides (25 out of 30 for EBV1 and 21 out of 30 for EBV2) and 13 out of 30 subjects reacted to the JCV peptide. (**Fig 2B and S2 Info**). These data for the positive control peptides of this second array experiment are comparable to those of the first array and to the described prevalence. Non-reactive *O*. *volvulus* peptides showed again very low reactivity, except for some outliers, indicating that these non-reactive *O*. *volvulus* peptides are also non-reactive in this healthy control population. The highly reactive *O*. *volvulus* peptides were mostly negative, but some peptides were found to be positive in one or more samples (**Fig 3B and S2 Info**).

### Evaluating inter-array reproducibility

The inter-array reproducibility between the 3.3M array and the 179K array was assessed by comparing the normalized profiles obtained in both experiments for six samples. Spearman correlation analysis revealed a high positive linear correlation between repeatedly analyzed peptides in five samples and moderate correlation in one sample (correlation coefficient range between 0.67 and 0.81 with *p*-value <0.001, **S1 Fig**).

### Identifying infection-specific antigenic peptides

For all peptides that were included in the 179K array, the number of positive samples (defined as signal >5,000 RLU) were determined both for subjects with a specific helminth infection (data from array experiment 1: *A*. *lumbricoides*: 10, *T*. *trichiura*: 11, hookworm: 9 and *S*. *mansoni*: 8) and healthy subjects (data from array experiment 2: 24) (All data available at https://osf.io/q5s9y/?view_only=8a464d9eafe6444b83b967a11f3211cd). Peptides for which at least one infected individual and not more than 1 out of 24 (i.e. ≤ 4.2%) healthy controls were positive (signal >5,000 RLU) were selected and the resulting set of peptides was sorted based on the Δ-value (= average response in the infected group—average response in healthy controls). For each infection, the 20 peptides with the highest Δ-value are listed in **Table 3.** Several peptides in this list were found to be strongly antigenic in several infection groups (e.g. ALUE_0000851001_mRNA_1;551). It is however not clear from the current data whether these peptides are truly pan-helminth markers or whether this is caused by the fact that several of the investigated individuals had multiple infections or had experienced prior exposure to the other STHs but without a patent infection at the moment of sample collection.

**Table 3:** Top 20 peptides identified for each infection. This table reports the 20 peptides with the highest Δ-values for each infection. The Δ-values were calculated by subtracting the average intensity (in relative light units (RLU)) in healthy controls (HC) from the average intensity (in RLU) in the infected group. The number of positive samples (i.e. signal > 5,000 RLU) for both the infected group (n = 10 for *A*. *lumbricoides*, n = 11 for *T*. *trichiura*, n = 9 for hookworm and n = 7 for *S*. *mansoni*) and the healthy control group (n = 24) is indicated for each of the peptides listed.

Species	Peptide ID	Uniprot Protein name	Peptide sequence	Number of positive samples (>5,000 RLU)		RLU Range Infected	RLU Range Healthy Controls	Δ (Avg. Infected–Avg. HC)
				Infected	Healthy controls			
*A*. *lumbricoides*	ALUE_0000851001_mRNA_1;551	Unknown	AKQDSFISNESTKLH	8	1	26–46837	101–5111	21908
	Smp_034080.1;353	Putative erythrocyte membrane protein	GIEAIKQNDESEFPNN	8	1	294–48867	31–8289	18297
	NECAME_06710;144	HEAT repeat protein	INESSMKQASLISNVM	7	1	19–49175	23–5893	15963
	ALUE_0001246601_mRNA_1;617	DNA-directed RNA polymerase III subunit RPC3	ERQLKMESIIANIEAD	6	0	37–44595	41–3581	15640
	ANCDUO_02559;123	Zinc finger, C3HC4 type	GIDEAVLRRTVDDLLT	9	0	2951–47823	30–4345	14554
	GS_23998;441	Unknown	LIERQLKMESIIANIE	6	0	20–41166	22–3109	14321
	Smp_152630.1;1	MEG-12	GENYEQQLQQPKAYGI	4	0	10–62200	22–1453	13232
	TTRE_0000850001_mRNA_1;188	205 kDa Pk1(B+)1+ SICAvar antigen	GVTANDLRTAEAMVRS	6	0	8–46446	19–571	13109
	NECAME_05279;46	Uncharacterized protein	MAKEDGLISNLRSYPP	7	1	49–41397	22–5999	13088
	Smp_063110.2;144	Putative zinc finger protein	GIEQQVILQPAAPVAM	6	0	240–40053	22–3087	12608
	NECAME_07428;155	PAP2_C domain-containing protein	GIEQLRPRELCGDLIV	7	0	962–34212	26–4284	12265
	GS_23620;782	Unknown	GIEEMTPSKKANNTAG	8	1	68–45073	28–8035	12252
	Smp_103170.1;78	Unknown	PPNYTPERYLEMGNRN	4	1	431–51442	188–8920	12195
	Smp_151380.1;507	Anaphase-promoting complex subunit 1	KTESLISNSPSTKKSK	6	0	9–40354	22–2015	12078
	ALUE_0000793101_mRNA_1;694	Unknown	GIEKIREKLNPFVVEK	5	1	182–51442	50–10302	11861
	NECAME_00764;3928	Uncharacterized protein	LKKIIGGHLQMPTAIV	5	0	6–57516	20–386	11605
	ANCDUO_18503;111	Uncharacterized protein	SKFDSVILNGYNIVGD	6	0	5–50886	20–895	11425
	TTRE_0000749301_mRNA_1;12	Unknown	KTVPPRKKDSEISNSV	6	0	91–32314	26–2676	11359
	GS_00707;166	Unknown	GIDRVIDAFRNAYTQV	4	0	19–33437	23–1963	11106
	NECAME_01953;144	Uncharacterized protein	KNLISAQLNQTATGQP	6	0	1131–39427	36–4993	10934
*T*. *trichiura*	ALUE_0000851001_mRNA_1;551	Unknown	AKQDSFISNESTKLH	10	1	4645–48005	101–5111	21749
	ANCDUO_02559;123	Zinc finger, C3HC4 type	GIDEAVLRRTVDDLLT	11	0	5567–47823	30–4345	21584
	Smp_034080.1;353	Putative erythrocyte membrane protein	GIEAIKQNDESEFPNN	10	1	294–38372	31–8289	21424
	NECAME_01953;144	Uncharacterized protein	KNLISAQLNQTATGQP	6	0	44–53181	18–2211	16910
	GS_00707;166	Unknown	GIDRVIDAFRNAYTQV	8	0	1356–39427	36–4993	15994
	NECAME_06710;144	HEAT repeat protein	INESSMKQASLISNVM	8	1	733–43011	23–5893	15854
	Smp_152630.1;1	MEG-12	GENYEQQLQQPKAYGI	5	0	8–61569	22–1453	15837
	ALUE_0001246601_mRNA_1;617	DNA-directed RNA polymerase III subunit RPC3	ERQLKMESIIANIEAD	7	0	646–45073	41–3581	14147
	GS_23620;782	Unknown	GIEEMTPSKKANNTAG	8	1	68–49508	28–8035	13550
	ANCDUO_21963;35	Uncharacterized protein	GVEHLARVNCVLWQND	7	0	485–46611	52–4998	13079
	TTRE_0000257301_mRNA_1;122	Unknown	GIDERLAHVWQKTTNA	6	1	1231–45116	59–10335	12956
	GS_23998;441	Unknown	LIERQLKMESIIANIE	7	0	440–40911	22–3109	12878
	Smp_063110.2;144	Putative zinc finger protein	GIEQQVILQPAAPVAM	9	0	240–34765	22–3087	12661
	ANCDUO_10263;496	Putative polyribonucleotide nucleotidyltransferase	GIDHVLDKMAVMLDRP	8	1	2039–40107	25–6745	12588
	ANCDUO_05567;1	Uncharacterized protein	MEIANGKEWKSDVRET	5	0	20–51748	23–2684	12323
	GS_00126;243	Unknown	GVLEMIKKYLINYEHM	5	1	7–50732	32–5119	12294
	NECAME_02686;166	Unknown	DKIVSEASRYKDMLQF	5	1	366–54645	60–7441	12170
	GS_08916;309	Unknown	GIDDIDEDELECILAN	7	1	3027–40053	33–8529	12008
	TTRE_0000898701_mRNA_1;67	Unknown	GTVYKLQEMKPSVVKE	4	0	4–51748	20–895	11972
	ALUE_0001542901_mRNA_1;23	Unknown	ITCKVTGRYTSALENL	6	0	26–60526	19–941	11783
Hookworm	Smp_034080.1;353	Putative erythrocyte membrane protein	GIEAIKQNDESEFPNN	8	1	3894–36306	31–8289	18206
	ANCDUO_02559;123	Zinc finger, C3HC4 type	GIDEAVLRRTVDDLLT	8	0	1097–37428	30–4345	17623
	ALUE_0000851001_mRNA_1;551	Unknown	AKQDSFISNESTKLH	5	1	26–44812	101–5111	13798
	GS_23620;782	Unknown	GIEEMTPSKKANNTAG	7	1	1542–34806	28–8035	11960
	ALUE_0002154501_mRNA_1;221	Uncharacterized protein	FKELANKLLNKQQLAE	6	1	932–52374	44–5966	11891
	ALUE_0001868101_mRNA_1;34	Unknown	SLAKKLFGSSAGLNDD	4	0	183–43591	30–1890	11671
	ANCDUO_21963;35	Uncharacterized protein	GVEHLARVNCVLWQND	7	0	485–30867	52–4998	11594
	GS_15548;34	Unknown	SLAKKLFGSSAGLNDD	4	0	183–43591	32–2488	11582
	GS_00707;166	Unknown	GIDRVIDAFRNAYTQV	5	0	1942–31701	36–4993	11247
	ALUE_0000733401_mRNA_1;298	Unknown	AKNGRQLYDNYIEARK	4	0	126–32809	27–3427	11236
	GS_08916;309	Unknown	GIDDIDEDELECILAN	7	1	1710–40053	33–8529	11231
	GS_01502;573	Unknown	KNGRQLYDNYIEARKR	4	0	78–40862	28–1451	10892
	ANCDUO_10263;496	Putative polyribonucleotide nucleotidyltransferase	GIDHVLDKMAVMLDRP	6	1	1227–40107	25–6745	10755
	ANCDUO_06401;122	Uncharacterized protein	GPWHQKLTAKKVAKSM	4	1	15–38687	20–5099	10624
	ANCDUO_05567;1	Uncharacterized protein	MEIANGKEWKSDVRET	3	0	9–51748	23–2684	10587
	ALUE_0001434301_mRNA_1;111	Unknown	QDILNNKALLGDEKAR	4	0	13–57516	19–941	10349
	TTRE_0000259701_mRNA_1;67	Unknown	ARAGLQGDLLGLNVLC	4	0	6–38452	19–2095	10332
	NECAME_02954;232	Beta-lactamase	VATKKLIGDQMLNRLQ	4	0	159–33821	23–863	10284
	TTRE_0000798101_mRNA_1;166	Unknown	ILKKHDKVLQNTAGNE	3	0	43–43168	19–2709	10235
	ANCDUO_24637;1	COesterase domain-containing protein	MTKPVKKTRWHQELSA	4	0	7–39427	21–3782	10169
*S*. *mansoni*	Smp_152630.1;1	MEG-12	GENYEQQLQQPKAYGI	6	0	4443–62200	22–1453	33784
	NECAME_01953;144	Uncharacterized protein	KNLISAQLNQTATGQP	5	0	262–53181	18–2211	32222
	TTRE_0000898701_mRNA_1;67	Unknown	GTVYKLQEMKPSVVKE	6	0	2496–51748	20–895	27482
	ALUE_0000851001_mRNA_1;551	Unknown	AKQDSFISNESTKLH	6	1	676–48005	101–5111	25208
	ANCDUO_19104;23	Sas10 domain-containing protein	GTVRKEMQKYSGESRG	6	0	1313–44286	27–2391	21927
	NECAME_06710;144	HEAT repeat protein	INESSMKQASLISNVM	5	1	180–43011	23–5893	20615
	NECAME_10074;1464	Uncharacterized protein	IELDPSAATSNTLHDV	4	0	85–62200	18–2359	20462
	Smp_162000.1;6582	UBR-type domain-containing protein	QKIAISEELNAKQINS	5	0	383–51442	28–1930	20002
	NECAME_00764;3928	Uncharacterized protein	LKKIIGGHLQMPTAIV	5	0	9–57516	20–386	19697
	ALUE_0001246601_mRNA_1;617	DNA-directed RNA polymerase III subunit RPC3	ERQLKMESIIANIEAD	4	0	146–45073	41–3581	19667
	TTRE_0000164301_mRNA_1;56	Unknown	FVVIENLSNDEKQIFT	5	0	176–41166	28–2467	19404
	TTRE_0000806301_mRNA_1;342	Unknown	ELELDPAKTNVNGGAI	5	0	179–60104	18–3029	19162
	ALUE_0001256701_mRNA_1;1255	Unknown	KKLISEALNSETRLQN	5	0	466–39540	27–3464	18956
	Smp_034080.1;353	Putative erythrocyte membrane protein	GIEAIKQNDESEFPNN	4	1	2764–39968	31–8289	17679
	ALUE_0000089701_mRNA_1;89	Uncharacterized protein	GTVLKFPSLSPFANAA	5	1	66–40220	26–6676	17505
	GS_00094;969	Unknown	FVVKEKRNANQFDVNN	4	1	445–42092	136–13481	17494
	TTRE_0000788501_mRNA_1;287	Unknown	YKHVIGIDLNKEAIEN	4	0	306–39209	26–2762	16791
	TTRE_0000483901_mRNA_1;89	Unknown	VSKQTISAHLKPLGSN	4	1	28–59047	22–6293	16727
	TTRE_0000134801_mRNA_1;243	Unknown	SPLIGKEIVSLSLNGD	4	0	31–46525	22–687	16646
	ANCDUO_07179;45	REX1 DNA Repair	IDREIVRILEEKLDHL	5	0	606–42756	27–1848	16589

### Conversion to peptide ELISA for MEG-12 peptide

**Fig 4:**
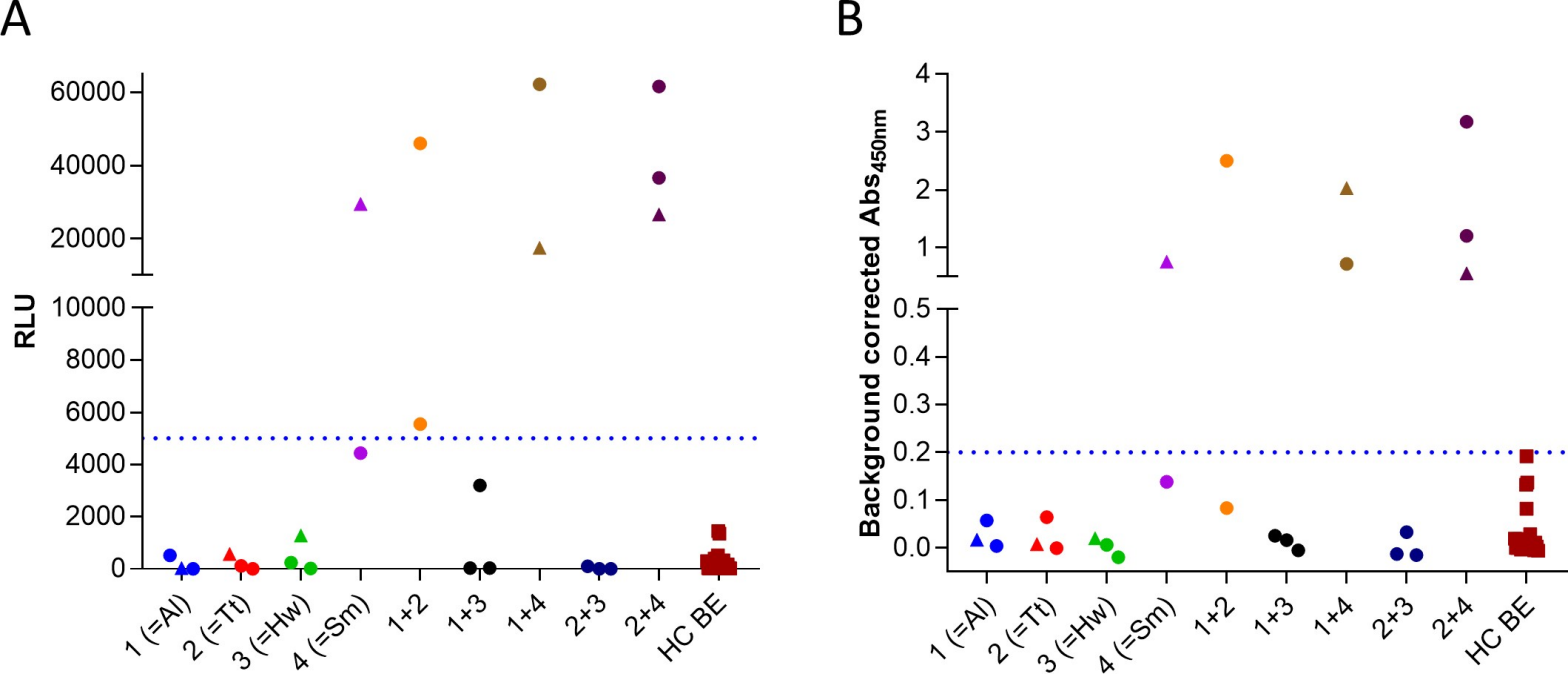
**Assessment of immune response against Smp_152630.1;1 using peptide array (A) and peptide ELISA (B).** Immunoreactivity against the MEG-12 peptide was determined in peptide ELISA on the same samples that were used in the arrays. Samples that were only measured in array 1 are indicated by circles, samples that were measured in both array 1 and 2 by triangles, and samples that were measured only in array 2 by squares. Dotted line indicates the cut-off for positivity. For the peptide ELISA this was defined as the signal that corresponds to 100% specificity.

The data presented in Table 3 indicated that one particular peptide in the *S*. *mansoni* protein MEG-12 (Smp_152630.1;1) appeared to be a promising candidate to detect infection with *S*. *mansoni*. In order to confirm these findings, a peptide ELISA was setup for this peptide and the same samples as used in the arrays were tested on this assay (**Fig 4B)**. The data obtained on the peptide ELISA were comparable to the data from the arrays (**Fig 4A)**. One *S*. *mansoni* infected individual was found to be negative, but just below the cut-off on both the array and the ELISA. One other individual that did not have a patent infection with *S*. *mansoni* (but with *A*. *lumbricoides* and *T*. *trichiura*) was found to have a strong immune response against this MEG-12 peptide in both formats. This latter individual was a 14-year old girl for which it can’t be excluded that she had ever been infected with *S*. *mansoni*.

## Discussion

The STH community identified non stool-based (e.g. serum and urine) biomarkers as the way forward to support programs in making the decision of whether or not to stop intervention efforts and to ultimately transit towards a sustainable post-intervention surveillance phase. In the current study, we aimed to identify a subset of linear antigenic peptides in the complete STH and *S*. *mansoni* proteome by performing two consecutive microarray experiments that could serve as potential biomarkers for helminth exposure in serum samples.

### Identification of antigenic linear peptides for specific STH species revealed to be sobering

Our approach existed of two subsequent peptide microarray experiments. The first experiment was developed to identify STH and *S*. *mansoni* peptides that are recognized by antibodies from children infected with one or more of these helminth infections. This allowed us to reduce the number of target peptides from 2,832,723 to 170,185. In the second part of the experiment, we were able to eliminate 27,034 peptides which elicited a detectable response in more than one out of the 24 healthy control sera. Eventually, the resulting set of peptides was sorted based on the Δ-value (average response in the infected group—average response in healthy controls) and the 20 peptides with the highest Δ-value were reported for each helminth species. However, results of both peptide arrays indicate that the identification of antigenic linear peptides for each STH species separately is not straightforward. A huge amount of overlap is seen between the peptides detected by the children identified to be infected with one or more STHs. The major reason for this is likely the fact that it is impossible to have information on the infection history of endemic individuals. We did have a high level of certainty on the type and level of the patent infections the children were carrying at the time of sampling by performing stool analysis using multiple different diagnostic methods (Kato-Katz, McMaster and Mini-FLOTAC). However, it remains impossible to tell what infections these children have been exposed to over their lifetime. In this respect, it has been described before that seroprevalence for STH appears to be higher than coproprevalence, further supporting this hypothesis [27]. It is very likely that these children have been exposed to a number of different endemic helminth species in the past. Even some helminth species which were not investigated or for which diagnosis was not performed (e.g. *Enterobius vermicularis* and *Strongyloides stercoralis*).

### Linear peptide of MEG-12 protein highly antigenic for *S*. *mansoni* infections

Although several peptides were found to be antigenic in multiple infection groups, the signal intensities of a particular peptide derived from the *S*. *mansoni* protein MEG-12 (for micro-exon gene 12) was notably higher in the *S*. *mansoni* infected group (Δ-value = 33,784; min. RLU = 4,443; max. RLU = 62,200), indicating a possible link with *S*. *mansoni* infection. The findings from the array were also confirmed in peptide ELISA. Interestingly, MEG-12 has been suggested as diagnostic marker for schistosome infection [44,45] and many of these MEGs have been shown to be immunogenic [46,47]. They have even been suggested as new group of targets that might be exploited for vaccine development [48]. Of interest, on position 100 of the top performing peptides in the array for *S*. *mansoni*, a peptide was found from MEG-2 (Smp_159800.1). Like MEG-12, also its N-terminal tail was found to be immunogenic in the array. MEG-2 is a protein that was found to be present in *Schistosoma* egg secretions, making it readily accessible for the immune system [49]. A more targeted evaluation of the serodiagnostic potential of these MEG proteins, either as recombinant proteins or as overlapping peptides, might be of interest for future studies. Recently, an epitope mapping study of a set of secreted or exposed proteins from the alimentary tract and tegument of *S*. *mansoni* identified exactly the same MEG-12 peptide as was detected in this study as one of the most highly immunogenic peptides and suggested it to be included in a multi-epitope vaccine construct [50]. The use of this epitope in a vaccine construct would however preclude the use of the same epitope in a serodiagnostic test. Increased emphasis on the coordination of both diagnostic and vaccine efforts would be essential to avoid vaccine-induced seroreactivity.

### There is a need for well-characterized biobanks for biomarker discovery

For further downstream analysis of the usefulness of the identified peptides as well as for future studies aimed at identifying serological biomarkers, serum samples are needed which are much better defined. This could be achieved through (i) using longitudinal samples from endemic subjects with changing infection status; (ii) using samples from young children living in areas which are endemic for only a single STH (e.g. *Ascaris*-only positive children from region A, *Trichuris* only positive children from region B, etc.); (iii) using sera from experimentally infected non-endemic individuals (e.g. experimental infections in man) [51,52] or (iv) by using sera from experimentally infected animals using the original or a closely related parasite species (e.g. hookworm infections in gerbils/dogs, *Schistosoma* spp. or *Trichuris muris* infections in mice, *A*. *suum* or *T*. *suis* infections in pigs [53–56]).

The importance of well-characterized biobanks will become even more wanted in the near future as countries will attempt to integrate control or monitoring programs of multiple NTDs [57]. Clear regulations on sample sharing and the design of well-formulated informed consent forms that allow for broader sample use would be very useful for future trials. A similar discussion on the need for well-characterized biobanks has also been made for *O*. *volvulus*. Several peptides were identified that were assumed to be linked to *O*. *volvulus* infection but were later found to be linked–at least partly—to other immunogens, possibly from parasitic origin [58].

### Limitations of linear peptide microarrays

Microarray results show that a great number of the peptides detected by the different infected populations actually originate form proteomes of other helminths. We can think of two explanations for this. First, some proteins are identical or have high degree of homology between the included helminth species. When constructing the 15-mer peptides to include onto the microarray, it is possible that for example a peptide that originates from the *A*. *lumbricoides* proteome also exists in identical or near identical form in the proteome of any of the other helminths. Second, it is so that, even though we measure the binding of antibodies to the different peptides on the microarray, this antibody affinity to these targets is not necessarily induced by that respective peptide or mother protein. Peptides could be recognized by antibodies that were initially targeting much different proteins carrying similar epitopes or mimotopes. The respective peptide thus acquires the structure of an (conformational) epitope that mimics the epitope that originally stimulated the immune system and antibody production. Our microarray approach focusses on linear epitopes. This has proven a successful approach for viral diseases like HIV and HCV [59–61]. However, our current data seem to indicate that it will not be as straightforward for helminth parasites [58]. It seems difficult to use antibody detection against linear epitopes as a good measure of helminth or parasite exposure. Maybe the use of other antibody Isotypes (IgG4) could reduce the level of cross-reactivity or be more indicative of active or recent infection (IgM). Alternatively, a set of peptides, either individually as a classifier, or as a multi-epitope construct, might be useful to cover the multitude of individual immune responses against these helminths.

### Conclusion

Based on the data presented here, it is unlikely that linear epitopes will serve to be highly useful in detecting species-specific antibody responses to STH in endemic communities. This study also emphasis the need of well-characterized biobanks for serological biomarker discovery, this is in particular when integration of multiple NTD programs is envisioned. The fact that our experiments resulted in limited sensitive and specific peptides that can act in multiplex technology with performance characteristics as described for use case 4 casts a doubt over the approach [12]. The developing timelines for STH and SCH peptide-based point-of-care diagnostics will most likely become cost-prohibitive. Instead, other (non-immune based) approaches might be more useful in this respect.

## Supporting information

S1 Fig Figure showing the correlation plots of the RLU values of the 6 samples included in both array experiment 1 and 2. (PNG)

S1 Info Excel file containing all data on the control and *Onchocerca volvulus* peptides included on microarray experiment 1. (XLSX)

S2 Info Excel file containing all data on the control and *Onchocerca volvulus* peptides included on microarray experiment 2. (XLSX)
