# Supplementary figures and images for: Identification of antigenic linear peptides in the soil-transmitted helminth and Schistosoma mansoni proteome
Source: PLoS Negl Trop Dis. 2021 Apr 28;15(4):e0009369. doi: 10.1371/journal.pntd.0009369 (PMC8081252; doi:10.1371/journal.pntd.0009369)

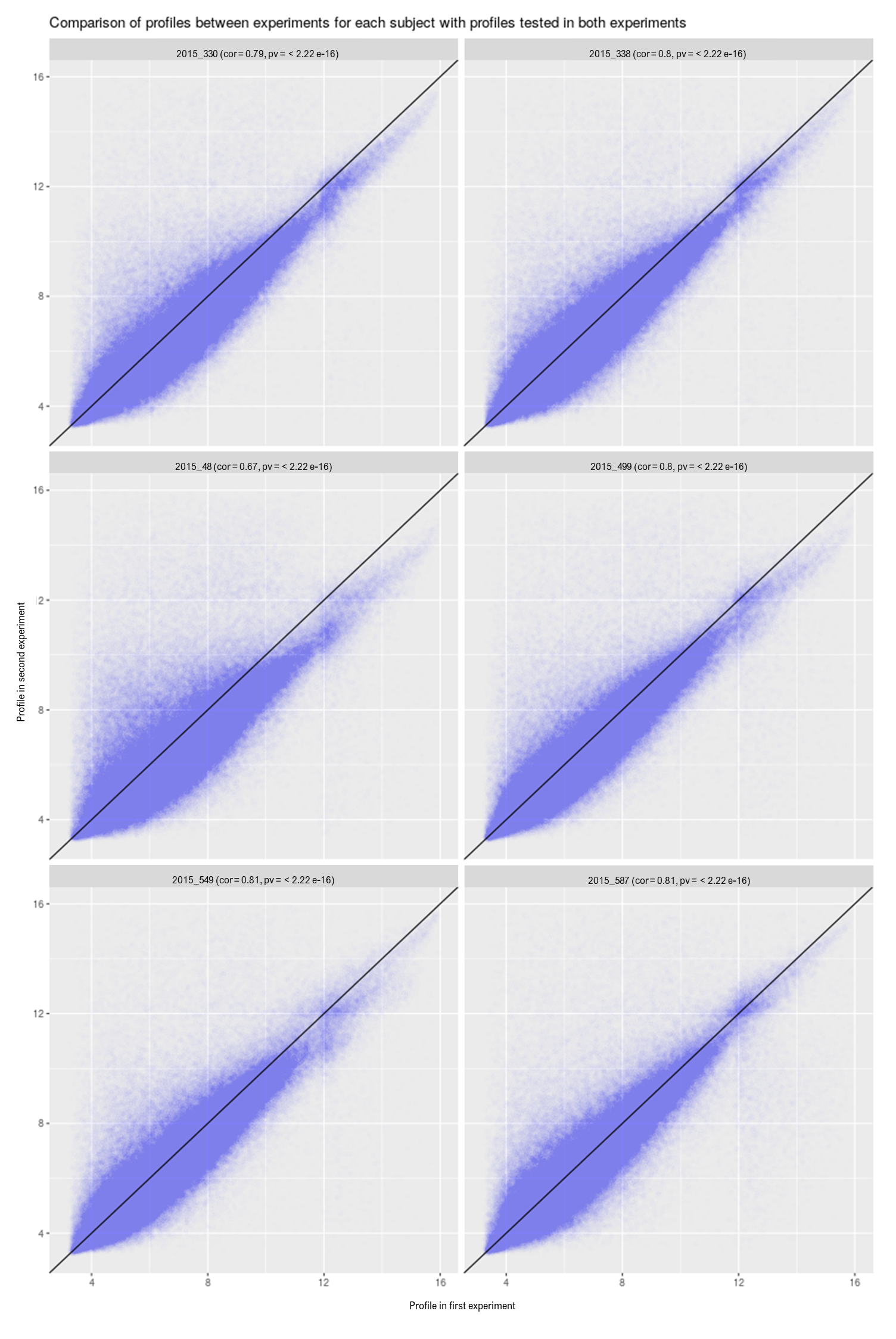

Supplement: S1 Fig — (PNG) [file pntd.0009369.s001.png]
